# Subgroup Invariant Perturbation for Unbiased Pre-Trained Model Prediction

**DOI:** 10.3389/fdata.2020.590296

**Published:** 2021-02-18

**Authors:** Puspita Majumdar, Saheb Chhabra, Richa Singh, Mayank Vatsa

**Affiliations:** ^1^ Department of Computer Science and Engineering, Indraprastha Institute of Information Technology, New Delhi, India; ^2^ Department of Computer Science and Engineering, Indian Institute of Technology Jodhpur, Rajasthan, India

**Keywords:** Fairness, trustability, bias estimation, bias mitigation, subgroup invariant perturbation, gender classification, race classification

## Abstract

Modern deep learning systems have achieved unparalleled success and several applications have significantly benefited due to these technological advancements. However, these systems have also shown vulnerabilities with strong implications on the fairness and trustability of such systems. Among these vulnerabilities, bias has been an *Achilles’ heel problem*. Many applications such as face recognition and language translation have shown high levels of bias in the systems towards particular demographic sub-groups. Unbalanced representation of these sub-groups in the training data is one of the primary reasons of biased behavior. To address this important challenge, we propose a two-fold contribution: a bias estimation metric termed as *Precise Subgroup Equivalence* to jointly measure the bias in model prediction and the overall model performance. Secondly, we propose a novel bias mitigation algorithm which is inspired from adversarial perturbation and uses the PSE metric. The mitigation algorithm learns a single uniform perturbation termed as *Subgroup Invariant Perturbation* which is added to the input dataset to generate a transformed dataset. The transformed dataset, when given as input to the pre-trained model reduces the bias in model prediction. Multiple experiments performed on four publicly available face datasets showcase the effectiveness of the proposed algorithm for race and gender prediction.

## 1. Introduction

Increasing use of artificial intelligence (AI) and machine learning (ML) for automation coupled with instances of biased predictions has motivated and mandated researchers across the globe to pursue designing dependable AI systems. Out of the several attributes of dependability in AI systems such as interpretability, explainability, robustness, bias, and fairness (Mehrabi et al., 2019; Drozdowski et al., 2020; Ntoutsi et al., 2020), this research is focused towards bias and fairness.

Face analysis tasks such as face detection, face recognition, expression analysis, age and gender prediction are some of the AI applications in which several instances of biased or unfair predictions have been observed. For instance, Buolamwini and Gebru (2018) have shown that commercial gender classifiers perform better for lighter skin males while giving poor performance for darker skin females. Other instances include false identification of 28 members (specifically people of color) of the US Congress as criminals by Amazon's facial recognition tool (Paolini-Subramanya, 2018). Nagpal et al. (2019) analyzed several pre-trained face recognition models to determine where and how the bias manifests in the deep neural networks. In light of these incidents, while some corporate and government organizations have decided to minimize or ban the development or usage of automated face analysis systems (Conger et al., 2019), several others are continuing the deployment and usage. Therefore, it is of paramount importance that we design mechanisms to improve the trustability and dependability of these systems. To address the challenges related to biased predictions of AI systems, researchers are broadly pursuing three directions: understanding bias, mitigating bias, and accounting for bias (Ntoutsi et al., 2020). Understanding bias involves realizing the source of bias along with estimating it (Buolamwini and Gebru, 2018; Celis and Rao, 2019; Nagpal et al., 2019; Radford and Joseph, 2020) whereas mitigation strategies involve designing algorithms that address bias (Creager et al., 2019; Nagpal et al., 2020).

In the literature, it has been demonstrated that if the training data used for learning the models is not balanced in terms of demographic subgroups, for instance, *male* and *female* are two different subgroups of *gender*, then there can be significant differences in the classification performance of pre-trained models observed on subgroups (Barocas and Selbst, 2016). Recent instances of biased predictions can be safely attributed to this observation as the training data required for deep learning models is often collected from the Internet using convenience sampling, which inherently leads to disparate proportions of data across subgroups. Models trained on historically biased datasets lead to biased results. Therefore, researchers have proposed several algorithms to mitigate the effect of bias on model prediction (Alvi et al., 2018; Gong et al., 2019). However, there is generally a trade-off between fairness and model performance (Du et al., 2019; Li and Vasconcelos, 2019). Removal of bias may affect the overall model performance while a high performing model may affect the performance of the under-represented subgroup. Therefore, it is important to 1) measure the trade-off between the effect of bias and the model performance through a unified metric and 2) mitigate the effect of bias without affecting the model performance. A solution to the problem is to re-train the models with large datasets having equal distribution of samples across different subgroups. However, in a real-world scenario, collecting such diverse datasets is not a trivial task. Also, re-training the models require updating millions of parameters and is computationally expensive.

**FIGURE 1:**
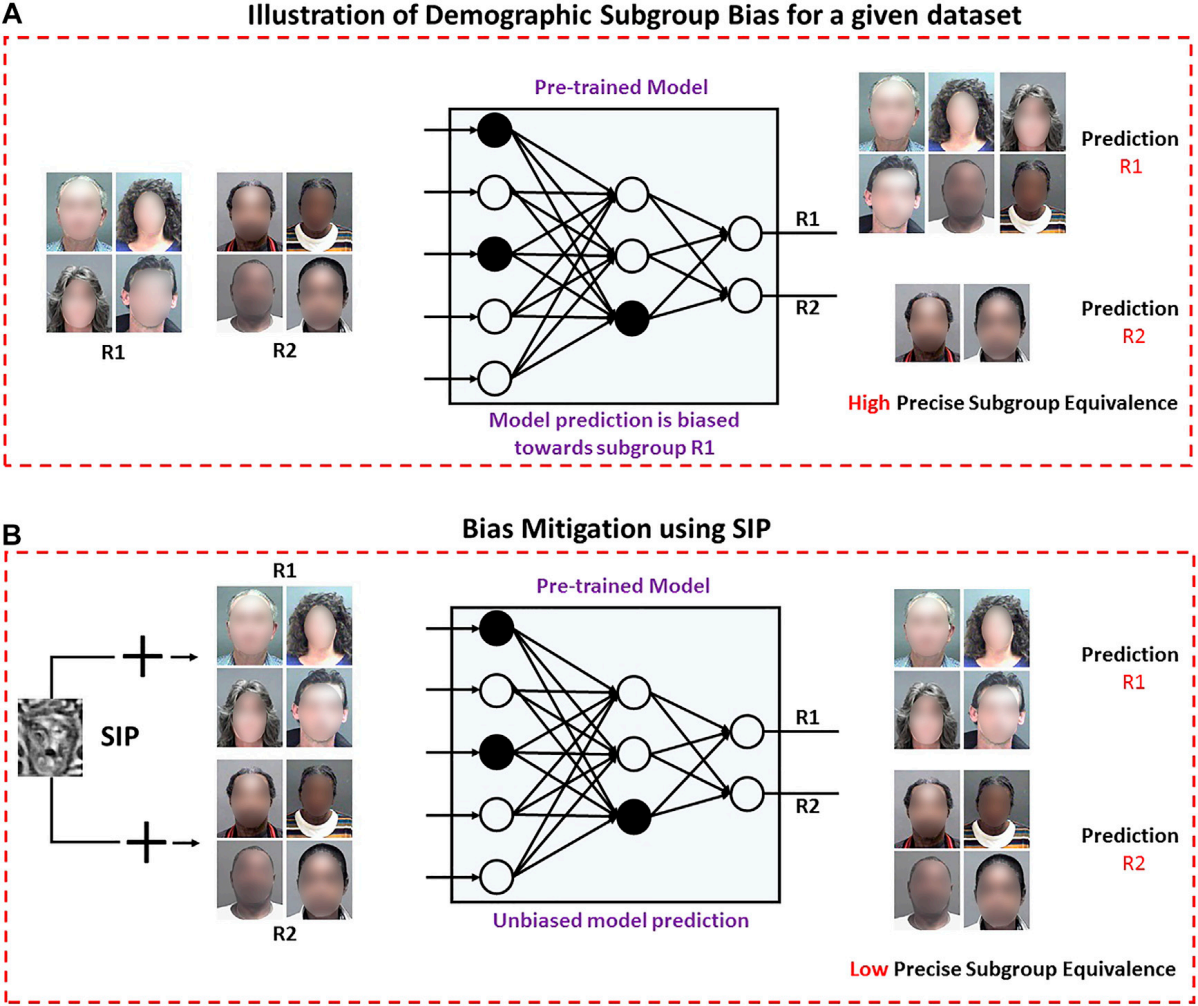
Effect of demographic subgroup bias on pre-trained model prediction. **(A)** Pre-trained model prediction is biased towards subgroup *R1*. **(B)** Bias mitigation using SIP to achieve equal performance across *R1* and *R2* (best viewed in color).

This research focuses on *estimating* the trade-off between the effect of bias and the model performance and *mitigating* the influence of demographic subgroup bias on pre-trained model prediction to improve the model performance. Existing metrics such as Disparate Impact, Average False Rate, and Degree of Bias provide information of only bias or error rates, but they do not provide the complete information. The first contribution of this research is a unified bias metric, termed as Precise Subgroup Equivalence (PSE) which provides a joint estimate of bias in model prediction and the overall model performance. The second contribution is to *mitigate* the influence of demographic subgroup bias on pre-trained model prediction to improve the model performance. We propose a novel algorithm based on adversarial perturbation for bias mitigation. In general, adversarial perturbation utilizes the vulnerability of deep models towards small changes in the input to reduce the confidence of model prediction. In this research, we have used this concept to reduce the effect of bias on model prediction. To the best of our knowledge, this is the first time that adversarial perturbation is used for bias mitigation. The proposed algorithm utilizes the model prediction to learn a single uniform Subgroup Invariant Perturbation (SIP) for a given dataset. SIP is added to the input dataset to generate a transformed dataset, which, when given as an input to the model, produces unbiased outcomes and improves the overall model performance. Figure 1 shows a visual illustration of the proposed algorithm for bias mitigation using SIP. The proposed algorithm is used to mitigate the impact of demographic subgroup bias in race and gender model predictions.

The effectiveness of the algorithm is demonstrated under two scenarios: 1) *independent demographic subgroup analysis* and 2) *inter-sectional demographic subgroup analysis* on multiple datasets to showcase enhanced performance and reduced effect of bias on model prediction. The results show that PSE provides a unified score of both error and disparity in subgroups which is addressed using the proposed algorithm. Further, since the number of learned parameters is equal to the size of the input image, the proposed algorithm is observed to be computationally efficient as well.

## 2. Related Work

Recent years have observed significant increase in the research on different aspects of bias and fairness in AI systems. Existing literature can be grouped into three broad categories: 1) Understanding and Estimating Bias, 2) Bias Mitigation Algorithms, and 3) Fairness Metrics.

Understanding and Estimating Bias: Researchers have focused on understanding the presence of bias in the prediction of commercial-off-the-shelf systems (COTS) and pre-trained deep models. Buolamwini and Gebru (2018) evaluated commercial gender classifiers from Microsoft, IBM, and Face ++ on four categories based on the skin type, namely, darker males, darker females, lighter males, and lighter females. It was found that the classifiers performed best for males with lighter skin tone and least for females with darker skin tone. Nagpal et al. (2019) provided an analysis of bias in deep face recognition models. They have shown that deep models encode race and age-specific features that lead to biased discrimination. According to various studies, the training data distribution has a huge impact on the model's performance (Torralba and Efros, 2011; Bolukbasi et al., 2016). Models trained on imbalanced datasets lead to biased outputs. Therefore, different data re-sampling techniques have been proposed by the researchers to balance the training data distribution. This is done either by over-sampling the minority class (Mullick et al., 2019) or under-sampling the majority class (Drummond et al., 2003). However, a recent study has shown that even models trained with balanced datasets amplify bias (Wang et al., 2019). It is shown that the learned models amplify the association between labels and gender, which in turn leads to biased discrimination.

Bias Mitigation: Mitigation algorithms can either be applied as a pre-processing step or in-processing, or post-processing. Different algorithms have been proposed to mitigate the effect of bias. Ryu et al. (2017) addressed the problem of the performance gap in different subgroups of race and gender attributes. They hypothesized that faces look different across different genders and races, and proposed InclusiveNet which learns the demographic information prior to attribute detection. Dwork et al. (2018) proposed decoupled classifiers to increase fairness and accuracy in classification systems. The decoupled classifiers learn a separate classifier for sensitive attributes and can be used with any black-box network. Das et al. (2018) proposed a Multi-Task Convolution Neural Network (MTCNN) to classify gender, age, and ethnicity attributes and minimized the effect of bias by utilizing disjoint features of fully connected layers of a deep Convolution Neural Network (CNN). Alvi et al. (2018) proposed a joint learning and unlearning framework for mitigating bias in CNN models for gender, age, race, and pose classification. A disentangled representation learning technique is presented to obtain flexibly fair features by Creager et al. (2019). Kim et al. (2019) proposed a regularization algorithm to unlearn the bias information. Recently, Nagpal et al. (2020) proposed a filter drop technique for learning unbiased representations. Results are demonstrated for gender prediction across different ethnicity groups.

Apart from bias mitigation in attribute prediction, researchers have also focused on mitigating bias in face recognition. Gong et al. (2019) addressed the problem of bias in face recognition systems and proposed a debiasing adversarial network. The proposed network learns unbiased representation for both identity and demographic attributes. Huang et al. (2019) investigated the problem of deep imbalanced learning in the context of deep representation learning for attribute prediction and face recognition. They proposed Cluster-based Large Margin Local Embedding (CLMLE) method, which maintains inter-cluster margin among the same and different classes. Wang and Deng (2019) proposed a reinforcement learning-based race balance network (RL-RBN) to mitigate racial bias. Singh et al. (2020) provided a review of techniques related to bias in face recognition.

Fairness Metrics: To measure the fairness of deep models, different metrics have been proposed in the literature.

Statistical Parity (SP) (Calders and Verwer, 2010): It is one of the widely used fairness metrics. It suggests that a model gives unbiased output if the prediction is independent of the demographic group such as *race*, *gender*, and *religion*. Deviation from statistical parity is measured as the ratio of the probability of a positive classification for both subgroups of a demographic group. It is termed as Disparate Impact (DI) (Feldman et al., 2015) and computed as:

$$DI=\frac{P\left(\hat{Y}=1|D=0\right)}{P\left(\hat{Y}=1|D=1\right)}$$ (1)

where, *D* represents the demographic group, and $\hat{Y}$ represents the predicted decision or class. A lower value of DI indicates higher bias in the model prediction.

Degree of Bias (DoB) (Gong et al., 2019): It is defined as the standard deviation of *Classification Accuracy* (*CAcc*) across different subgroups of a demographic group. Mathematically, it is represented as:

$$DoB=std\left(CAcc_{D_{j}}\right)\;\forall j$$ (2)

where, $D_{j}$ represents a subgroup of a demographic group *D*. High performance gap across different subgroups will result in high DoB, which in turn implies bias in the model prediction.

## 3. Materials and Methods

The following subsections discuss the proposed metric, estimation of bias in model prediction, and bias mitigation using Subgroup Invariant Perturbation (SIP). There are two different scenarios for bias estimation and mitigation: 1) *independent demographic subgroup analysis* and 2) *intersectional demographic subgroup analysis*. In the first scenario, bias estimation/mitigation is performed across the subgroups of a demographic group. For example, bias estimation/mitigation is performed across the subgroups of *gender*. In the second scenario, bias estimation/mitigation is performed across the intersection of different demographic groups. For example, bias estimation/mitigation is performed across the intersectional subgroups of *race* and *gender*.

### 3.1. Proposed Metric: Precise Subgroup Equivalence

Existing fairness metrics evaluate the performance gap across different subgroups (Du et al., 2020). However, these do not reflect the overall model performance. For instance, if a model gives almost equal but low performance across different subgroups, then DI will be high, and DoB will be low. Therefore, the model prediction will be considered unbiased across different subgroups. However, an unbiased but low performing model is undesirable. Therefore, in this research, Precise Subgroup Equivalence (PSE) metric is introduced that jointly estimates the effect of demographic subgroup bias on model prediction and the overall model performance. Precise Subgroup Equivalence (PSE) is the average of Disparate Impact (DI), Average False Rate (AFR), and Degree of Bias (DoB).

$$PSE=\frac{\left(1-DI\right)+AFR+DoB}{3}$$ (3)

Since a lower value of DI indicates higher bias in model prediction, therefore higher value of (1−DI) indicates higher bias in model prediction. Here, AFR is the mean of *False Positive Rate* (*FPR*) and *False Negative Rate* (*FNR*). It is robust to the subgroup imbalance problem and reflects the overall model performance. On the other hand, (1−DI) and DoB reflects the bias in the model prediction. Therefore, PSE provides a joint estimate of the overall model performance and the impact of bias. A model with low PSE indicates an unbiased high performing model.

### 3.2. Bias Estimation

For joint estimation of pre-trained model performance and the impact of demographic subgroup bias, PSE of the model prediction corresponding to a given dataset is computed. Let X be the training set with *n* number of images.

$$X=\left\{X_{1},X_{2},\ldots .,X_{n}\right\}$$ (4)

where, each image $X_{i}$ is associated with *m* demographic groups. Let D and E are the two demographic groups and *s* and *t* be the number of subgroups in D and E, respectively.

$$D=\left\{D_{1},D_{2},\ldots .,D_{s}\right\}\;\text{and}\;E=\left\{E_{1},E_{2},\ldots .,E_{t}\right\}$$ (5)

where, $D_{j}$ and $E_{j}$ represent a subgroup of the respective demographic group. Let $\phi_{D}$ be a pre-trained model with weight W and bias *b* trained for predicting demographic group D.

For the first scenario, the probability of predicting an input image $X_{i}$ to subgroup $D_{j}$ is represented as:

$$P\left(D_{j}|X_{i},D\right)=\phi_{D}\left(X_{i},W,b\right)$$ (6)

For the second scenario, the probability of predicting an input image $X_{i}$ to subgroup $D_{j}$ across demographic group E is represented as:

$$P\left(D_{j}|X_{i},E,D\right)=\phi_{D}\left(X_{i},W,b\right)$$ (7)

The PSE of model $\phi_{D}$ corresponding to dataset X is computed as:

$$PSE_{\phi_{D}}=\frac{\left(1-DI_{\phi_{D}}\right)+AFR_{\phi_{D}}+DoB_{\phi_{D}}}{3}$$ (8)

where, $DI_{\phi_{D}}$, $AFR_{\phi_{D}}$, and $DoB_{\phi_{D}}$ are the Disparate Impact, Average False Rate, and Degree of Bias of model $\phi_{D}$ corresponding to dataset X, respectively.

### 3.3. Bias Mitigation

After estimating the bias in the prediction of a pre-trained model $\phi_{D}$ corresponding to dataset X, the next task is to mitigate the effect of bias to improve the overall model performance. For this purpose, a single uniform Subgroup Invariant Perturbation (SIP) is learned by minimizing the PSE corresponding to the first scenario for the given dataset X. The aim is to generate a transformed dataset T by adding SIP to all the images of dataset X, such that when T is given as input to the pre-trained model $\phi_{D}$ produces unbiased outcomes and improves the overall performance. We hypothesize that the learned SIP is effective for mitigating the bias corresponding to the second scenario as well. In order to validate this, multiple experiments are performed, and the results are discussed in Section 5.2. The optimization process for learning SIP N is discussed below.

Let N be the Subgroup Invariant Perturbation (SIP), initialized with zeros. Each image $X_{i}$ of the dataset has pixel values in the range of {0,1}. Let T be the transformed dataset obtained by adding N to the dataset X. To bring the pixel values of each image in the transformed dataset in the range of {0,1}, tanh function is applied as follows:

$$T_{i}=\frac{1}{2}\left(tanh\left(X_{i}+N\right)+1\right)$$ (9)

where, $T_{i}$ represents the transformed image corresponding to the input image $X_{i}$. The probability of predicting a transformed image $T_{i}$ to subgroup $D_{j}$ is given by:

$$P\left(D_{j}|T_{i},D\right)=\phi_{D}\left(T_{i},W,b\right)$$ (10)

**FIGURE 2:**
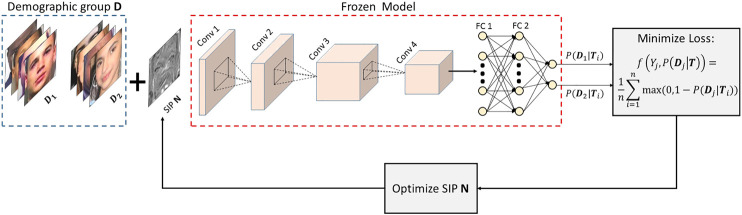
Block diagram of the steps involved in learning Subgroup Invariant Perturbation (SIP). In the first step, SIP N is initialized with zeros and added to the images of the training set to generated the transformed set. In the next step, the transformed set is given as input to the pre-trained model and model prediction is obtained. Next, loss is computed and optimization is performed over N to minimize PSE. The updated N is added to the training set and the process is repeated until convergence (best viewed in color).

For models that yield biased predictions, there is a performance gap across different subgroups, where the performance of some subgroups are better than others. Therefore, the objective is to reduce PSE by 1) enhancing the performance of the low performing subgroups and 2) maintaining/enhancing the performance of high performing subgroups. In order to achieve both the objectives, the following objective function is used.

$$f\left(Y_{i,j},P\left(D_{j}|T_{i},D\right)\right)$$ (11)

where, $Y_{i,j}$ represents the true label and f(.,.) is the function to minimize the distance between the true label and the probability of predicting the true class. The above objective function is optimized corresponding to SIP N. For this purpose, the following function is minimized:

$$\underset{N}{\text{min}}\;f\left(Y_{i,j},P\left(D_{j}|T_{i},D\right)\right)\;\;\forall j$$ (12)

$$f\left(Y_{j},P\left(D_{j}|T,D\right)\right)=\frac{1}{q}\overset{q}{\underset{i=1}{\sum }}max\left(0,1-P\left(D_{j}|T_{i},D\right)\right)$$

where, j∈{1,…,s} and *q* is the number of images belonging to subgroup *j* with q<n. f(.,.) will increase the probability of predicting the true class, which in turn reduces the $PSE_{\phi_{D}}$. Low $PSE_{\phi_{D}}$ will simultaneously ensure reduced effect of bias on model prediction along with improved model performance. Figure 2 shows the block diagram of the steps involved in learning the SIP N.

## 4. Experimental Setup

The performance of the proposed algorithm is evaluated for race and gender classification on four different datasets. The results are reported using the proposed metric PSE, two existing bias evaluation metrics, and one existing performance evaluation metric. The details of the datasets with the corresponding protocols and the pre-trained models used for the experiments are discussed below.

### 4.1. Databases and Protocols

**FIGURE 3:**
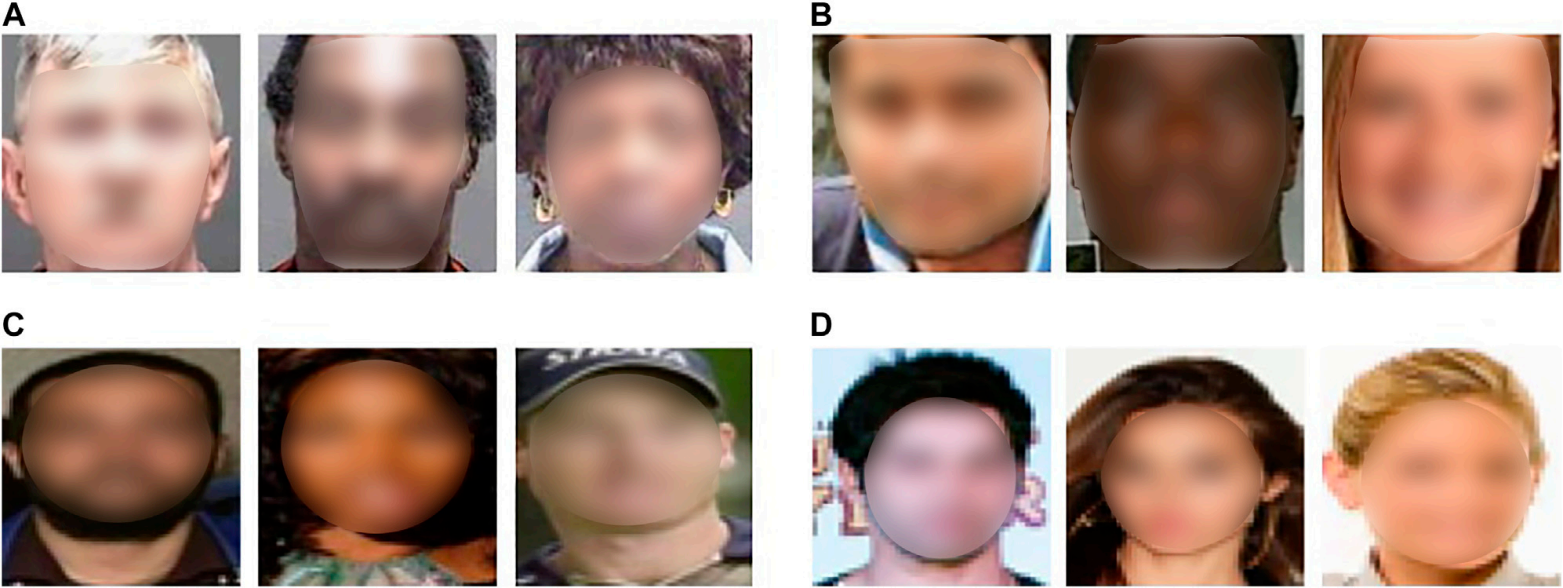
Sample images of the **(A)** MORPH, **(B)** UTKFace, **(C)** LFWA, and **(D)** CelebA datasets.

Experiments are performed for race and gender prediction, using data corresponding to race *R1 (light skin color)* and *R2 (dark skin color)*, and gender *G1 (Male)* and *G2 (Female)*. The distribution of the number of images in each dataset across different race and gender subgroups is shown in Table 1; Figure 3 shows sample images from each dataset.

**TABLE 1:** Distribution of number of images in the MORPH, UTKFace, LFWA, and CelebA datasets across different race and gender subgroups.

Dataset	Race		Gender	
	*R*1	*R*2	*G*1	*G*2
MORPH	10,662	42,725	46,835	8,527
UTKFace	10,076	4,525	12,389	11,312
LFWA	9,830	560	10,181	2,962
CelebA	—	—	75,976	1,06,756

MORPH dataset (Album-2) (Rawls and Ricanek, 2009) contains more than 54,000 images of 13,180 subjects. The dataset is partitioned into 60% training set, 20% validation set, and 20% testing set. The partitioning is done with non-overlapping subjects in each set.

UTKFace dataset (Zhang et al., 2017) contains more than 20,000 face images and divided into three parts, having 9,779, 10,718, 3,206 images in Part I, Part II, and Part III, respectively. Part I is used for training, Part II for testing, and Part III for validation (Das et al., 2018).

LFWA dataset (Huang et al., 2008) contains 13,233 images of 5,749 subjects with 73 attributes. Attributes corresponding to each image is annotated with intensity values. These are binarized by converting positive intensity values with label 1 and negative intensity values with label 0. For experiments, attributes corresponding to race *R1*, *R2*, and gender *G1* are taken. Images with label 0 for *G1* are considered as *G2*. Experiments are performed using the standard pre-defined protocol proposed by (Huang et al., 2008).

CelebA dataset (Liu et al., 2015) consists of a total of 2,02,599 face images of more than 10,000 celebrities with 40 annotated binary attributes. For experiments, the *G1* attribute is taken and images with label 0 for *G1* are considered as *G2*. The experiments are performed using the standard pre-defined protocol defined by (Liu et al., 2015).

Pre-trained models: Experiments are performed using pre-trained VGGFace (Parkhi et al., 2015) model, which is trained on the VGGFace dataset (Parkhi et al., 2015) for face recognition. VGGFace dataset is a large scale dataset of 2.6M facial images corresponding to 2.6K subjects. VGGFace model has shown high generalization abilities for face recognition. Therefore, we have used this model and fine-tuned it for race and gender prediction. In this research, three race prediction models and four gender prediction models are used for the experiments. The race prediction models are obtained by separately fine-tuning the pre-trained VGGFace model on the MORPH, UTKFace, and LFWA datasets. Similarly, the gender prediction models are obtained by fine-tuning on the MORPH, UTKFace, LFWA, and CelebA datasets. These models are treated as pre-trained race and gender prediction models in all the experiments.

### 4.2. Implementation Details

The implementation details of the network training and perturbation learning for mitigation are given below.

Network training: Each model is trained by adding two fully connected dense layers of 512 dimensions after the final convolutional layer of the VGGFace model. Models are trained for 20 epochs with Adam optimizer. The learning rate is set to 0.0001 for the first 10 epochs and reduced by 0.1 after every 5 epochs. Categorical cross-entropy loss is used to train the models.

Perturbation learning for mitigation: Perturbation is learned from the training set of a given dataset. In order to learn Subgroup Invariant Perturbation (SIP), a matrix is initialized with zeros of size 64×64×3 (equal to the dimension of the input image), which results in 12,288 number of parameters. The parameters of this matrix are only trainable during SIP learning while keeping the parameters of the model frozen. In the first step, SIP is added to the images in the training set using Equation 9 and given as input to the model to obtain the predictions. In the second step, model predictions are used to compute the loss using Equation 12. In the final step, the gradient of the loss is computed with respect to the given input, and this gradient is backpropagated to the input to update the parameters of the SIP matrix only. The process is repeated until convergence. For perturbation learning, Adam optimizer is used with a learning rate of 0.001. Depending upon the training set, the batch size is set between 500 and 1,000. Each batch is processed for 16 iterations.

## 5. Results and Analysis

Models trained on datasets with over-representation of some demographic subgroups and under-representation of others often result in biased outputs. In a real-world scenario, it is difficult to have knowledge of the dataset used to train a model. However, depending on the training data distribution, the model could lead to biased prediction outputs. Therefore, it is important to first estimate the bias in model prediction, followed by mitigation. As discussed previously, the model's overall performance should also be considered during the estimation/mitigation of bias in model prediction to balance the trade-off between fairness and model performance. Therefore, in this research, we have jointly estimated bias in model prediction and the overall model performance using the proposed metric PSE. A series of experiments are performed where models pre-trained on some datasets are evaluated on others for bias estimation using the existing and proposed metrics. Next, we use the PSE of the model to mitigate the effect of bias in model prediction using the proposed algorithm.

We have segregated this section into: 1) Bias Estimation and 2) Bias Mitigation in Sections 5.1 and Sections 5.2, respectively. Analysis of the experiments are performed under both the scenarios, *Independent demographic subgroup analysis* and *Intersectional demographic subgroup analysis*. In the first scenario of independent demographic subgroup analysis, bias estimation/mitigation algorithms are analyzed across the subgroups of a demographic group individually. Whereas, in the second scenario, analysis is performed across the intersection of different demographic groups. Table 2 shows the details of the experiments performed in this research.

**TABLE 2:** Details of the experiments to estimate and mitigate the effect of demographic subgroup bias on pre-trained race and gender prediction models.

Task	Scenario	Model trained on	Bias estimation/mitigation
Race prediction	Independent/intersectional demographic subgroup analysis	MORPH	UTKFace, LFWA
		UTKFace	MORPH, LFWA
		LFWA	MORPH, UTKFace
Gender prediction	Independent demographic subgroup analysis	MORPH	UTKFace, LFWA, CelebA
		UTKFace	MORPH, LFWA, CelebA
		LFWA	MORPH, UTKFace, CelebA
		CelebA	MORPH, UTKFace, LFWA
	Intersectional demographic subgroup analysis	MORPH	UTKFace, LFWA
		UTKFace	MORPH, LFWA
		LFWA	MORPH, UTKFace

### 5.1. Bias Estimation

Bias estimation plays a key role in designing solutions for bias mitigation. Therefore, it is important to have a good metric to estimate bias in model prediction along with the overall model performance. There are various fairness and performance evaluation metrics, such as DI, DoB, and AFR. DI measures the deviation from statistical parity, and DoB represents the standard deviation of classification accuracy across different subgroups. On the other hand, AFR gives the average of the false positive rate and false negative rate. These metrics either evaluate the performance gap across different subgroups or the overall model performance. Therefore, we have introduced a new metric PSE that evaluates both fairness and model performance. To validate this fact, we have evaluated the performance of multiple pre-trained models (trained on different datasets) using existing and proposed metrics. The experimental setup of this experiment is discussed below:

Experimental Setup: In this experiment, the performance of pre-trained models is evaluated using five different evaluation metrics: subgroup-specific error rate, (1-DI), DoB, AFR, and PSE for bias estimation. Evaluation of each pre-trained model is done on the training set of all the datasets except the one on which the model is trained. For instance, if the model is pre-trained on the MORPH dataset, then it is evaluated on the LFWA, CelebA, and UTKFace datasets. This setup is considered by keeping in mind the real-world scenario where the training set of the pre-trained model is unknown. Bias estimation is done on the training set because the PSE learned from the training set is used to mitigate the bias in model prediction for the corresponding dataset.

#### 5.1.1. Independent Demographic Subgroup Analysis

In this scenario, the models are evaluated across different *race* and *gender* subgroups, respectively, of a given dataset. The error rate of each subgroup is computed to understand the variations in performance across subgroups. Table 3 shows the performance of pre-trained race prediction models. It is observed that the error rate of the models varies significantly across different *race* subgroups. It is also observed that the distribution of training data plays a significant role in the performance of pre-trained models. For instance, the model trained on the MORPH dataset when evaluated on the UTKFace dataset results in 27.72% and 22.47% error rate corresponding to subgroup *R*1 and *R*2, respectively. On the other hand, when the LFWA model is evaluated on the UTKFace dataset, it gives 0.04% and 97.54% error rate corresponding to subgroup *R*1 and *R*2, respectively. The significant difference in the error rate of each subgroup obtained by different pre-trained models is due to the skewed training data distribution on which these models are trained as shown in Table 1. The MORPH dataset has under-representation of subgroup *R*1 and over-representation of subgroup *R*2. On the other hand, the LFWA dataset has a majority of subgroup *R*1. Therefore, the model trained on the MORPH dataset performs better for subgroup *R*2, while the LFWA model gives better performance for subgroup *R*1. A similar observation can be drawn when the evaluation is performed on the LFWA dataset using the models trained on the MORPH and UTKFace datasets.

**TABLE 3:** Performance of pre-trained *race* prediction models (%) across different *race* subgroups for independent demographic subgroup analysis scenario.

Bias estimated on	Model trained on	Error		1 – DI	AFR	DoB	PSE
		R1	R2				
UTKFace	MORPH	27.72	22.47	6.77	25.09	2.62	11.49
	LFWA	0.04	97.54	97.53	48.78	48.75	65.02
MORPH	UTKFace	0.52	80.02	79.92	40.27	39.75	53.31
	LFWA	0.00	96.86	96.86	48.43	48.43	64.57
LFWA	MORPH	83.32	7.64	81.94	45.48	37.84	55.08
	UTKFace	17.82	60.37	51.78	39.09	21.27	37.38

On evaluating the performance of a pre-trained model using individual metrics for a given dataset, it is observed that PSE is a good indicator of fairness and model performance. For instance, the PSE of the LFWA model corresponding to the UTKFace dataset is 65.02%. The high value of PSE indicates a biased and low performing model. The values of metrics (1-DI) and DoB are 97.53% and 48.75%, indicating a biased model. However, these do not provide any insights about model performance. On the other hand, the AFR of the model is 48.78%, indicating that the model performance is low without providing any insight about the bias in model prediction. This shows that metric PSE provides a joint estimation of bias and model performance.

The performance of the *gender* prediction models is reported in Table 4. A similar observation is drawn regarding the effectiveness of metric PSE from Table 4. For instance, the performance of the model trained on the UTKFace dataset, when evaluated on the MORPH dataset, shows almost equal but high error rate across different *gender* subgroups. Therefore (1-DI) and DoB of this model are low, but AFR is high. Thus, none of the metrics is able to provide a unified estimate of fairness and model performance. On the other hand, the PSE of this model is 13.86% showing a joint estimate of both. A similar observation is obtained when this pre-trained model is evaluated on the LFWA and CelebA datasets. This showcases that PSE provides a unified score of both error and disparity in subgroups.

**TABLE 4:** Performance of pre-trained *gender* prediction models (%) across different *gender* subgroups for independent demographic subgroup analysis scenario.

Bias estimated on	Model trained on	Error		1 – DI	AFR	DoB	PSE
		G1	G2				
UTKFace	MORPH	29.42	42.75	18.90	36.08	6.66	20.54
	LFWA	24.94	49.12	32.22	37.02	12.09	27.11
	CelebA	53.05	41.70	19.47	47.37	5.67	24.17
MORPH	UTKFace	36.75	33.53	4.85	35.13	1.61	13.86
	LFWA	39.05	24.96	18.77	32.00	7.04	19.27
	CelebA	69.82	29.78	57.01	49.79	20.02	42.27
LFWA	UTKFace	30.27	36.69	9.21	33.48	3.21	15.30
	MORPH	19.27	57.66	47.56	38.46	19.19	35.07
	CelebA	16.79	45.74	34.80	31.26	14.47	26.84
CelebA	UTKFace	43.23	42.88	0.62	43.05	0.17	14.61
	MORPH	39.71	57.79	30.00	48.75	9.04	29.26
	LFWA	12.21	54.75	48.45	33.47	21.27	34.40

#### 5.1.2. Intersectional Demographic Subgroup Analysis

Existing studies (Alvi et al., 2018; Das et al., 2018; Nagpal et al., 2020) have shown that the influence of one demographic group can affect the prediction of others. For instance, the performance of a gender prediction model may be affected due to the imbalance in ethnicity subgroups. In such a case, the model prediction will be biased towards the over-represented ethnicity subgroup. Therefore, it is important to estimate the bias of one demographic group on the prediction of others. For this purpose, in this scenario, the pre-trained *race* prediction models are evaluated across different *gender* subgroups and vice versa. This scenario showcases the performance of the pre-trained models across the intersection of different demographic groups.

Table 5 shows the results of this experiment. On evaluating the performance across all the datasets using different pre-trained race prediction models, it is observed that the models trained on the UTKFace and LFWA datasets result in a high error rate for predicting race *R*2 across *G*2, i.e., subgroup (*R*2, *G*2). It is also observed that none of the samples in this intersectional subgroup are correctly classified by the model trained on the LFWA dataset when evaluated on the UTKFace dataset. This results in a high PSE value of 65.83%. For gender prediction across *race* subgroups, it is observed that all the pre-trained *gender* prediction models (except model trained on the LFWA dataset when evaluated on the MORPH dataset) perform worse for predicting gender *G*2 across *R*2, i.e., subgroup (*G*2, *R*2). The results from Table 5 highlight that the majority of the pre-trained *race* and *gender* prediction models do not perform well for (*R*2, *G*2) and (*G*2, *R*2) subgroups, respectively.

**TABLE 5:** Performance of pre-trained *race* prediction models (%) across different *gender* subgroups and *gender* prediction models across *race* subgroups of a given dataset for intersectional demographic subgroup analysis scenario.

Bias estimated on	Model trained on	Error				1 – DI	AFR	DoB	PSE
		G1		G2					
		R1	R2	R1	R2				
Race prediction across gender subgroups									
UTKFace	MORPH	35.70	18.41	20.76	26.47	14.21	25.33	5.74	15.09
	LFWA	1.15	97.52	1.30	100.00	98.75	49.98	48.76	65.83
MORPH	UTKFace	0.56	79.71	0.36	81.95	80.74	40.64	40.18	53.85
	LFWA	0.00	96.55	0.00	98.74	97.64	48.82	48.82	65.09
LFWA	UTKFace	20.49	58.96	9.52	67.40	56.17	39.09	24.08	39.78
	MORPH	83.24	6.99	83.58	10.87	81.78	46.17	37.23	55.06
Gender prediction across race subgroups									
UTKFace	MORPH	32.07	38.95	16.42	54.91	28.09	35.58	11.34	25.00
	LFWA	28.97	43.68	9.46	62.75	39.79	36.21	16.99	30.99
MORPH	UTKFace	36.76	11.05	36.85	41.81	18.38	31.61	7.66	19.22
	LFWA	44.50	17.80	37.70	27.66	23.19	31.91	9.18	21.43
LFWA	UTKFace	30.70	35.28	31.01	50.00	17.08	36.75	5.89	19.91
	MORPH	19.65	57.28	17.47	58.70	48.39	38.27	19.71	35.46

### 5.2. Bias Mitigation

The experiments performed for bias estimation show that the pre-trained models do not give equal performance across different subgroups. Therefore, in this experiment, a single uniform Subgroup Invariant Perturbation is learned by minimizing the PSE of the pre-trained model prediction to achieve improved and almost equal performance across different subgroups. Multiple experiments are performed to evaluate the effectiveness of the proposed algorithm to mitigate the effect of bias in pre-trained model prediction. As mentioned in Section 3.3, SIP learned corresponding to the ‘independent subgroup analysis’ scenario is used to evaluate the performance of the proposed algorithm for the ‘intersectional subgroup analysis’ scenario as well. The performance of the proposed algorithm is compared with pre-trained and fine-tuned model predictions. Performance is evaluated using multiple existing metrics and the proposed metric PSE. Additionally, we have compared the number of trainable parameters of the proposed algorithm with model fine-tuning. Experimental setup of this experiment is discussed below:

Experimental Setup: In this experiment, SIP is learned corresponding to the training set of all the datasets individually other than the dataset on which the pre-trained model is trained. The learned SIP is added to the testing set of the corresponding dataset for evaluating the performance of the proposed algorithm. For instance, the model pre-trained on the MORPH dataset learns SIP using the training set of the UTKFace dataset and bias is estimated on the testing set of the UTKFace dataset. Similarly, during bias estimation of the MORPH model on the LFWA dataset, SIP learned on the training set of the LFWA dataset is used. For fine-tuning, the pre-trained model is updated using the training set of a given dataset and evaluated on the testing set of the corresponding dataset. The performance of the pre-trained model is evaluated on the testing set of the corresponding dataset.

#### 5.2.1. Independent Demographic Subgroup Analysis

The results of the pre-trained model, fine-tuned model, and the proposed mitigation algorithm are summarized in Table 6. It is observed that the proposed algorithm reduces the bias in the model prediction and enhances the performance. For instance, the proposed algorithm reduces the PSE by 8.99% and 21.39% from the pre-trained and fine-tuned MORPH model predictions, respectively, for the UTKFace dataset. It is interesting to observe that fine-tuning increases the bias in the model prediction and decreases the overall performance. This is because the fine-tuned model decreases the error rate from 33.95 to 4.85% of subgroup *R*1 but increases the error rate of subgroup *R*2 from 18.61 to 46.37% compared to the pre-trained model. The UTKFace dataset has an under-representation of subgroup *R*2. Therefore, a model fine-tuned on this dataset decreases the error rate of subgroup *R*1 and penalizes subgroup *R*2. A similar observation can be drawn from the subgroup-specific error rates of fine-tuned MORPH and UTKFace models on the LFWA dataset, due to the minority of subgroup *R*2. On the other hand, the proposed algorithm overcomes the problem and reduces the performance gap across different subgroups.

**TABLE 6:** Performance of *race* prediction models (%) after bias mitigation using the proposed and existing algorithms [Multi-task (Das et al., 2018) and Filter Drop (Nagpal et al., 2020)] for independent demographic subgroup analysis scenario.

Bias estimated on	Model trained on		Error		1 – DI	AFR	DoB	PSE
			R1	R2				
UTKFace	MORPH	Pre-trained	33.95	18.61	18.86	26.27	7.67	17.60
		Fine-tuned	4.85	46.37	43.63	25.61	20.76	30.00
		Multi-task	29.66	13.32	18.85	21.48	8.17	16.17
		Filter drop	27.95	16.16	14.06	22.04	5.90	14.00
		Proposed	14.55	19.74	6.08	17.17	2.59	**8.61**
	LFWA	Pre-trained	0.08	97.45	97.45	48.76	48.68	64.96
		Fine-tuned	3.55	53.44	51.72	28.49	24.94	35.05
		Multi-task	31.55	14.50	19.93	23.02	8.53	17.16
		Filter drop	26.78	14.84	14.01	20.80	5.97	13.59
		Proposed	15.42	25.16	11.52	20.29	4.87	**12.23**
MORPH	UTKFace	Pre-trained	0.31	86.45	86.41	43.37	43.07	57.62
		Fine-tuned	6.04	1.85	4.27	3.94	2.09	3.43
		Multi-task	3.32	5.99	2.75	4.65	1.34	2.91
		Filter drop	4.13	5.76	1.69	4.93	0.82	2.48
		Proposed	2.47	4.91	2.51	3.68	1.22	**2.47**
	LFWA	Pre-trained	0.00	98.11	98.11	49.05	49.05	65.40
		Fine-tuned	7.09	1.73	5.46	4.41	2.68	4.18
		Multi-task	2.07	7.96	6.01	5.00	2.95	4.65
		Filter drop	3.22	6.54	3.42	4.87	1.66	3.32
		Proposed	1.31	4.46	3.20	2.88	1.57	**2.55**
LFWA	UTKFace	Pre-trained	19.85	63.86	54.92	41.85	22.00	39.59
		Fine-tuned	0.71	81.76	81.63	41.23	40.52	54.46
		Multi-task	32.04	24.92	9.49	28.47	3.56	13.84
		Filter drop	35.62	28.78	9.61	32.19	3.42	15.07
		Proposed	8.56	22.11	14.83	15.33	6.77	**12.31**
	MORPH	Pre-trained	81.95	2.11	81.56	42.02	39.92	54.50
		Fine-tuned	1.47	78.25	77.93	39.86	38.39	52.06
		Multi-task	32.61	27.02	7.66	29.81	2.80	13.42
		Filter drop	32.69	26.32	8.64	29.50	3.19	13.78
		Proposed	8.75	23.16	15.80	15.95	7.20	**12.98**

The lowest PSE value is highlighted.

**FIGURE 4:**
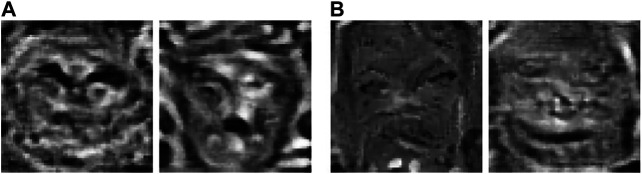
Visualization of the learned Subgroup Invariant Perturbation (SIP) corresponding to the **(A)** race and **(B)** gender prediction models.

The performance of *gender* prediction models is shown in Table 7. It is observed that the proposed algorithm reduces the PSE of each model corresponding to all the datasets. For instance, the PSE of the pre-trained and fine-tuned UTKFace model corresponding to the MORPH dataset is 22.91% and 17.53%, respectively. The proposed algorithm reduces the PSE to 8.34%. This showcase that the proposed algorithm is jointly able to reduce the bias in model prediction and improve the overall performance of the model. Figure 4 shows the visualization of the learned Subgroup Invariant Perturbation (SIP). A face like structure can clearly be seen in all the perturbations.

**TABLE 7:** Performance of *gender* prediction models (%) with the proposed and existing bias mitigation algorithms for independent demographic subgroup analysis scenario.

Bias estimated on	Model trained on		Error		1 – DI	AFR	DoB	PSE
			*G*1	*G*2				
UTKFace	MORPH	Pre-trained	22.64	41.37	24.56	32.13	9.36	22.02
		Fine-tuned	15.61	29.30	16.23	22.45	6.84	15.17
		Multi-task	23.65	37.87	18.62	30.75	7.11	18.83
		Filter drop	28.08	34.99	9.60	31.52	3.46	14.86
		Proposed	10.18	22.30	13.50	16.24	6.06	**11.93**
	LFWA	Pre-trained	18.25	51.74	40.97	34.99	16.74	30.90
		Fine-tuned	15.00	31.67	19.62	23.33	8.33	17.09
		Multi-task	24.01	38.52	19.09	31.26	7.25	19.20
		Filter drop	29.77	33.33	5.06	31.54	1.78	12.79
		Proposed	11.91	23.15	12.77	17.53	5.62	**11.97**
	CelebA	Pre-trained	42.96	47.32	7.65	45.13	2.18	18.32
		Fine-tuned	15.14	33.44	21.57	24.28	9.15	18.33
		Multi-task	30.86	38.08	10.43	34.46	3.61	16.17
		Filter drop	29.12	34.93	8.19	32.01	2.91	14.37
		Proposed	34.70	34.98	0.44	34.83	0.14	**11.80**
MORPH	UTKFace	Pre-trained	41.27	19.02	27.48	30.14	11.12	22.91
		Fine-tuned	5.84	28.52	24.09	17.17	11.34	17.53
		Multi-task	10.00	22.80	14.22	16.39	6.40	12.34
		Filter drop	8.88	21.67	14.03	15.27	6.40	11.90
		Proposed	14.11	3.59	10.92	8.84	5.26	**8.34**
	LFWA	Pre-trained	53.80	20.58	41.84	37.19	16.61	31.88
		Fine-tuned	8.69	18.75	11.02	13.72	5.03	9.92
		Multi-task	9.63	23.07	14.87	16.34	6.72	12.64
		Filter drop	9.88	20.94	12.26	15.4	5.53	11.06
		Proposed	15.92	4.75	11.72	10.33	5.58	**9.21**
	CelebA	Pre-trained	66.47	31.45	51.09	48.95	17.51	39.18
		Fine-tuned	7.71	22.01	15.50	14.85	7.15	12.50
		Multi-task	8.19	24.60	17.87	16.39	8.21	14.16
		Filter drop	9.80	26.47	18.48	18.12	8.33	14.98
		Proposed	19.49	3.26	16.78	11.37	8.11	**12.09**
LFWA	UTKFace	Pre-trained	29.88	39.39	13.57	34.63	4.75	17.65
		Fine-tuned	3.99	54.18	52.28	29.08	25.09	35.48
		Multi-task	27.46	36.70	12.73	32.07	4.62	16.47
		Filter drop	30.16	34.05	5.56	32.09	1.95	13.20
		Proposed	18.12	26.58	10.33	22.35	4.23	**12.30**
	MORPH	Pre-trained	19.12	58.50	48.69	38.30	19.69	35.56
		Fine-tuned	5.50	50.42	47.53	27.95	22.46	32.65
		Multi-task	39.67	28.74	15.34	34.20	5.47	18.34
		Filter drop	34.43	37.32	4.40	35.86	1.45	13.90
		Proposed	15.95	27.33	13.55	21.63	5.69	**13.62**
	CelebA	Pre-trained	16.31	45.21	34.53	30.76	14.45	26.58
		Fine-tuned	10.56	36.79	29.33	23.67	13.11	22.04
		Multi-task	25.41	37.41	16.07	31.40	6.00	17.82
		Filter drop	25.44	34.72	12.49	30.08	4.64	15.74
		Proposed	12.61	28.02	17.64	20.31	7.70	**15.22**
CelebA	UTKFace	Pre-trained	42.62	42.77	0.27	42.69	0.07	14.34
		Fine-tuned	28.26	11.97	18.51	20.11	8.14	15.59
		Multi-task	—	—	—	—	—	—
		Filter drop	—	—	—	—	—	—
		Proposed	33.90	33.92	0.04	33.91	0.00	**11.32**
	MORPH	Pre-trained	38.67	56.47	29.02	47.56	8.90	28.49
		Fine-tuned	23.85	14.26	11.19	19.05	4.79	11.68
		Multi-task	—	—	—	—	—	—
		Filter drop	—	—	—	—	—	—
		Proposed	14.99	21.73	7.28	18.08	3.37	**9.58**
	LFWA	Pre-trained	11.71	55.06	49.10	33.38	21.67	34.72
		Fine-tuned	26.03	12.94	15.04	19.48	6.54	13.69
		Multi-task	—	—	—	—	—	—
		Filter drop	—	—	—	—	—	—
		Proposed	11.57	23.33	13.31	17.45	5.88	**12.21**

The lowest PSE value is highlighted.

**FIGURE 5:**
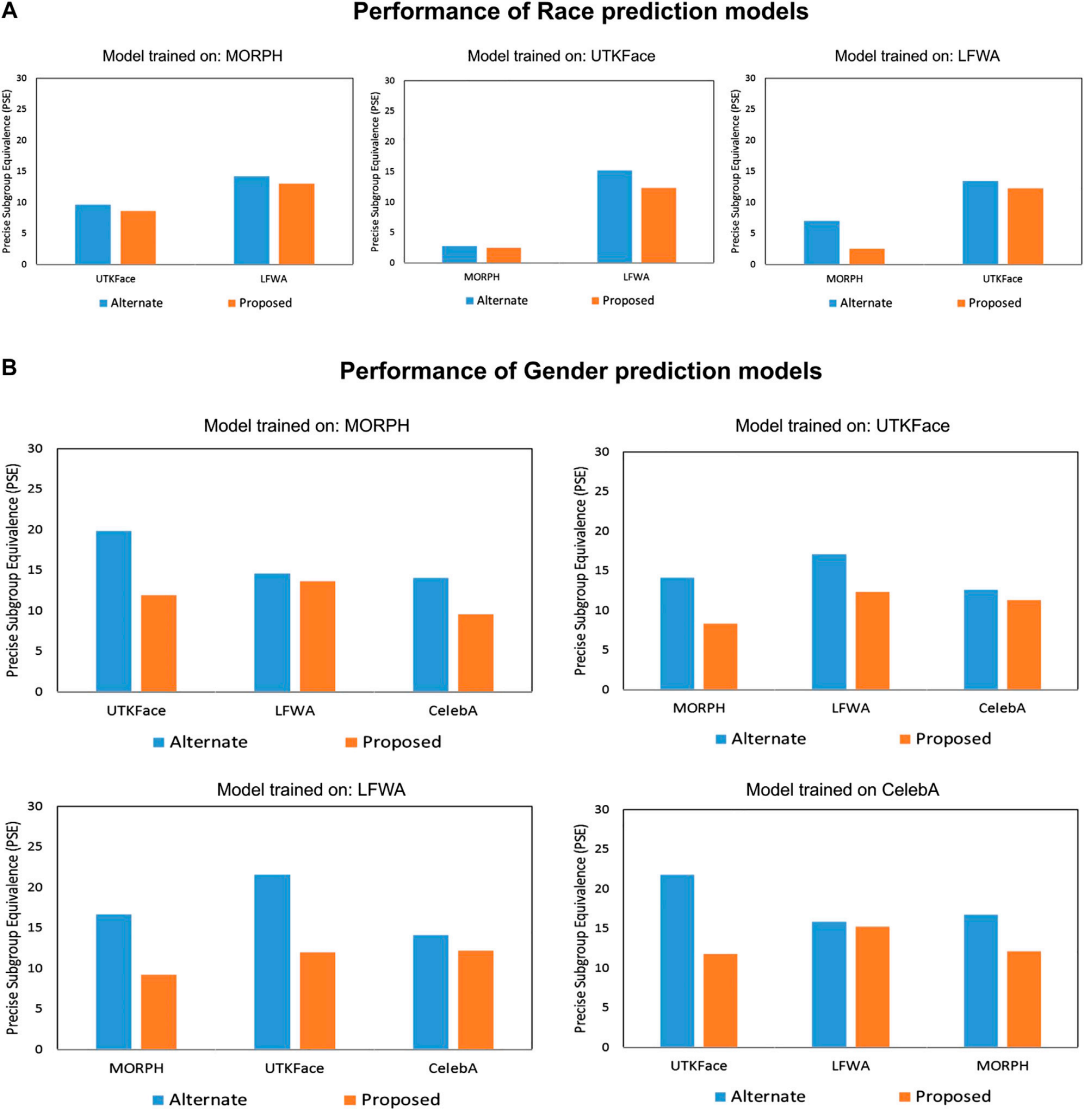
Results shows the comparison of alternate and joint optimization using the proposed algorithm corresponding to the independent demographic subgroup analysis scenario. The proposed algorithm gives the lowest PSE.

The proposed algorithm is compared with two existing bias mitigation algorithms (Das et al., 2018; Nagpal et al., 2020). The comparison of the results for *race* and *gender* prediction are shown in Tables 6 and 7, respectively. It is observed that the proposed algorithm outperforms existing algorithms for both *race* and *gender* prediction. The proposed algorithm jointly optimizes bias and the overall model performance while the existing algorithms focus on bias optimization only. Therefore, the PSE of the proposed algorithm is minimum compared to others. For instance, in *gender* prediction (Table 7), the PSE of the CelebA model corresponding to the UTKFace dataset for Multi-task (Das et al., 2018), Filter Drop (Nagpal et al., 2020), and the proposed algorithm is 16.17%, 14.37%, and 11.80%, respectively. This shows the effectiveness of the proposed algorithm for independent demographic subgroup analysis scenario. In our experimental setup, the existing algorithms are not applicable for *gender* prediction on the CelebA dataset. Apart from this, we have also performed an experiment, where we reduce bias and improve the model performance alternatively using the proposed bias mitigation algorithm. The results of this experiment are compared with the proposed bias mitigation algorithm, where we jointly reduce the bias and improve the model performance. Figure 5 shows the comparison of the results of alternate and joint optimization corresponding to the independent demographic subgroup analysis scenario. It is observed that joint optimization leads to better results as it provides combined supervision of bias and model performance for better learning of SIP that results in better performance.

#### 5.2.2. Intersectional Demographic Subgroup Analysis

**FIGURE 6:**
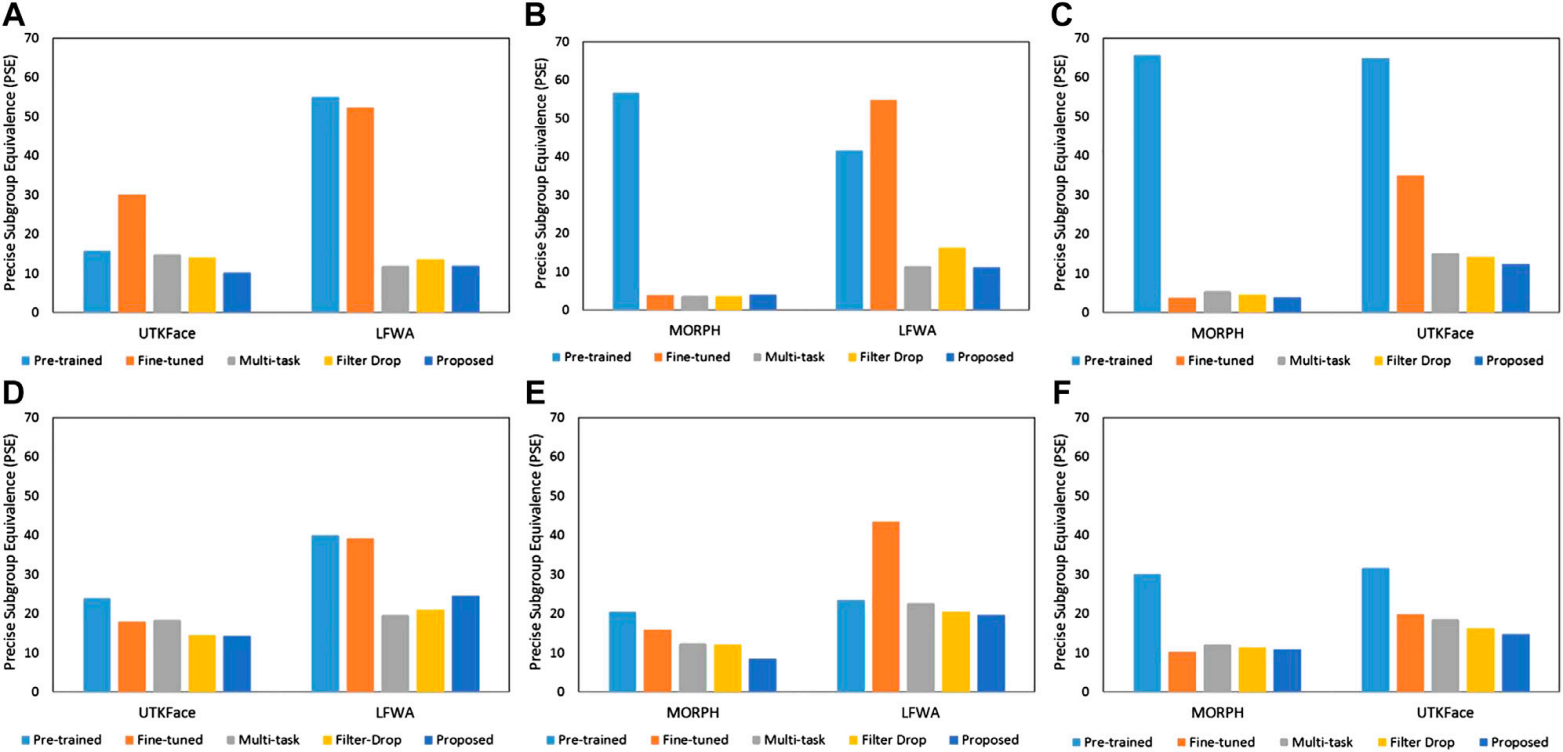
Results of **(A**–**C)** race classification across *gender* subgroups corresponding to the MORPH, UTKFace, and LFWA models, respectively and **(D**–**F)** gender classification across *race* subgroups corresponding to the MORPH, UTKFace, and LFWA models, respectively for intersectional demographic subgroup analysis scenario. Comparison is shown with pre-trained and fine-tuned model predictions along with existing algorithms for bias mitigation.

To further evaluate the effectiveness of the proposed algorithm across the intersection of different demographic groups, two different experiments are performed. In the first experiment, race classification is performed across *gender* subgroups. While in the second experiment, gender classification is performed across *race* subgroups. These experiments are performed to analyze the presence of *gender* bias on race prediction and *race* bias on gender prediction. Comparison is performed with pre-trained and fine-tuned model predictions. Figure 6 shows the PSE corresponding to the first and second experiments. It is observed that in most of the cases, the proposed algorithm gives the lowest PSE. For instance, the PSE of pre-trained and fine-tuned UTKFace models corresponding to the MORPH dataset for gender prediction is 20.58% and 15.91%, respectively. The proposed algorithm reduces the PSE to 8.56%. This indicates that the proposed algorithm is able to reduce the effect of bias of one demographic group on the prediction of others. The reduction in PSE shows the effectiveness of the proposed algorithm.

**FIGURE 7:**
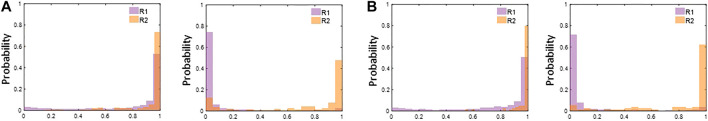
Score distribution of the MORPH race prediction model across *gender* subgroups **(A)** *G*1 and **(B)** *G*2 on the LFWA dataset. The first graph of **(A)** and **(B)** shows the score distribution of the pre-trained model and the second graph shows the score distribution of the proposed algorithm.

**FIGURE 8:**
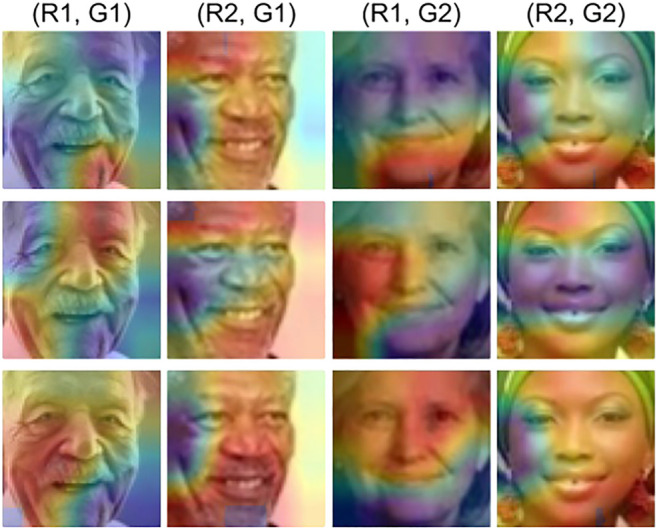
Class Activation Map of race classification across *gender* subgroups on the UTKFace dataset using the MORPH race prediction model. Top row shows the visualization for the pre-trained model prediction, middle row for the fine-tuned model prediction, and the bottom row for the proposed algorithm. It is observed that the proposed algorithm focuses on the entire facial region instead of the subgroup-specific region for feature extraction.

Figure 7 compares the performance of the proposed algorithm and the pre-trained model using the score distribution of the model prediction. The results are shown for *race* prediction across different *gender* subgroups of the MORPH model on the LFWA dataset. It is observed that the proposed algorithm reduces the overlap among the subgroups and separates them from each other. Class Activation Map (CAM) of race classification across *gender* subgroups on the UTKFace dataset using the MORPH race prediction model is shown in Figure 8. It is observed that the pre-trained and fine-tuned models focus on different facial regions across the intersection of different demographic subgroups. On the other hand, the proposed algorithm tries to focus on the entire facial region irrespective of different subgroups. This showcases the effectiveness of the learned SIP to mitigate the effect of demographic subgroup bias by enforcing the model to extract features from the entire facial region for discrimination instead of subgroup-specific regions.

On comparing the number of trainable parameters of the proposed algorithm with model fine-tuning, it is observed that the proposed algorithm requires number of parameters equal to the size of the input image, i.e., 12K parameters. On the other hand, model fine-tuning requires updation of 0.52M parameters, which is approximately 43 times more than the proposed algorithm. This shows that the proposed algorithm is computationally efficient.

Figure 6 shows the comparison of the proposed algorithm with existing bias mitigation algorithms (Das et al., 2018; Nagpal et al., 2020). It is observed that in most of the cases, the proposed algorithm performs better than existing algorithms while giving comparable results for others. For instance, the PSE of the proposed and existing algorithms for *race* prediction across *gender* subgroups of the MORPH model corresponding to the UTKFace and LFWA datasets are 10.24%, 14.83%, 14.10% and 12.06%, 11.94%, 13.57%, respectively. It is important to note that the proposed algorithm does not require model training and therefore is computationally efficient.

## 6. Discussion and Conclusion

The effect of demographic subgroup bias on the performance of commercial and pre-trained models is studied in the past. A lot of progress is made towards estimating and mitigating the influence of bias on model prediction. However, studies have shown that there is a trade-off between fairness and model performance. Maintaining a balance between the two is an important factor. This motivated us to propose a unified metric to measure the trade-off and an algorithm to mitigate the effect of bias on pre-trained model prediction.

We used multiple pre-trained race and gender prediction models for bias estimation and mitigation. Since the existing metrics either evaluate the performance gap across different subgroups or the overall model performance, therefore we have introduced a unified metric, PSE, to jointly estimate the bias in model prediction and the overall model performance. Additionally, a novel algorithm is proposed to mitigate the effect of bias using adversarial perturbation by reducing the PSE of the model prediction. We showed that a single uniform Subgroup Invariant Perturbation (SIP), when added to the input images, is able to mitigate the effect of bias on model prediction.

During bias estimation, it is observed that PSE reflects both error and disparity in subgroups. On analyzing the existing metrics, it is observed that DI and DoB do not reflect the overall model performance, while AFR does not reflect the performance gap across different subgroups. On the other hand, we have experimentally validated in Tables 3–5 that PSE considers the model performance along with fairness. Therefore, PSE is utilized by the proposed algorithm to learn SIP for bias mitigation. The performance of race and gender prediction models corresponding to the independent demographic subgroup analysis scenario are summarized in Tables 6 and 7, respectively. We have found that the proposed algorithm is able to reduce the PSE of all the pre-trained models corresponding to all the datasets. To test the proposed algorithm for mitigating the influence of bias corresponding to the intersectional subgroup analysis scenario, SIP learned corresponding to the independent subgroup analysis scenario is used. Figure 6 shows that the proposed algorithm is effective in mitigating the intersectional subgroup bias. This is validated by the score distributions in Figure 7 that shows that the proposed algorithm reduces the overlap between subgroups. We have also found that the proposed algorithm focuses on the entire face for feature extraction instead of subgroup-specific regions in Figure 8.

Existing research towards bias mitigation requires model training to suppress the element of bias for unbiased prediction. However, the proposed algorithm does not require model training for bias mitigation. It requires the number of trainable parameters equal to the size of the input image, which is significantly lower than the model fine-tuning approach. Therefore, the proposed algorithm is computationally efficient. This showcase the applicability of the proposed algorithm in real-world scenarios.

In the future, we plan to extend the proposed algorithm for mitigating the effect of bias due to the influence of multiple demographic subgroups via learning a single Subgroup Invariant Perturbation (SIP). Also, we will investigate the effect of bias on face recognition performance.

## Data Availability

Publicly available datasets were analyzed in this study. This data can be found here: MORPH: https://ebill.uncw.edu/C20231_ustores/web/store_main.jsp?STOREID=4, UTKFace: https://susanqq.github.io/UTKFace/, LFWA: http://vis-www.cs.umass.edu/lfw/, CelebA: http://mmlab.ie.cuhk.edu.hk/projects/CelebA.html.
